# Adolescents’ Assessment of Two Mental Health–Promoting Mobile Apps: Results of Two User Surveys

**DOI:** 10.2196/40773

**Published:** 2023-01-06

**Authors:** Helene Høgsdal, Sabine Kaiser, Henriette Kyrrestad

**Affiliations:** 1 Regional Centre for Child and Youth Mental Health and Child Welfare - North UiT The Arctic University of Norway Tromsø Norway

**Keywords:** mental health app, mobile app, mental health, mental health promotion, cyberbullying, adolescents, user satisfaction, system usability, app quality, mental health intervention, health promotion, app usability, user experience

## Abstract

**Background:**

The importance of mental health promotion is irrevocable and is especially important at a young age. More mental health-promoting mobile apps have been developed in the last few years. However, their usability and quality have been rarely assessed.

**Objective:**

The aim of this study is to investigate how adolescents assess the usability, quality, and potential goal achievement of Opp and NettOpp. Opp is a universal mental health–promoting mobile app aimed at 13- to 19-year-olds, and NettOpp is a mobile app for children and adolescents between 11 to 16 years of age that have experienced negative incidents online.

**Methods:**

A total of 45 adolescents tested either Opp (n=30) or NettOpp (n=15) for a period of 3 weeks and answered a questionnaire. The System Usability Scale (SUS) was used to measure the usability of the apps. A SUS score above 70 indicates acceptable usability. Items from the Mobile Application Rating Scale were adapted for study purposes and used to measure the quality and perceived goal achievement that Opp and NettOpp might have on adolescents’ knowledge, attitudes, and intention to change behavior. Furthermore, adolescents could answer an open comment question.

**Results:**

Opp had a mean SUS score of 80.37 (SD 9.27), and NettOpp’s mean SUS score was 80.33 (SD 10.30). In the overall evaluation, Opp and NettOpp were given a mean score of 3.78 (SD 0.42) and 4.20 (SD 0.56), respectively, on a 5-point scale, where 5 was best. Most adolescents who evaluated Opp rated that the app would increase knowledge about mental health and help young people deal with stress and difficult emotions or situations. Most adolescents who evaluated NettOpp agreed that the app would increase awareness and knowledge about cyberbullying, change attitudes toward cyberbullying, and motivate them to address cyberbullying. Some adolescents stated that Opp was difficult to navigate and consisted of too much text. Some of the adolescents that tested NettOpp stated that the app had crashed and that the design was a bit childish.

**Conclusions:**

All in all, this study indicates that Opp and NettOpp have good usability and that adolescents are satisfied with both apps. It also indicates that the potential goal achievement of the apps, for example, increasing knowledge about mental health (Opp) or cyberbullying (NettOpp) is promising. While there are some comments from the users that are more difficult to solve (eg, Opp is too text-based), some comments helped improve the apps (eg, that the app crashed). Overall, the user evaluation provided valuable knowledge about how adolescents assess Opp and NettOpp. However, more extensive effectiveness studies are necessary to measure their actual goal achievement.

## Introduction

### Background

Adolescence is a crucial time in life to develop healthy habits that can improve both physical and mental health in later life [1]. Moreover, evidence suggests that adolescents who are affected by mental health issues at an early age often experience negative consequences, such as mental disorders, in later adult life [2]. This makes it important to work toward ensuring adolescents have good conditions for a healthy upbringing [3]. However, there are many young people who are struggling. Prevalence rates show that around 10%-20% of the world’s youth population is affected by mental health problems [4]. It is therefore important to develop and implement effective preventive tools for adolescents. In today’s digital world, it has become increasingly popular to offer young people preventive interventions through mobile apps to improve health outcomes (ie, mobile health apps) [5].

In general, mental health promotion aims at increasing resilience, self-esteem, and coping skills [3]. Traditionally, most of the mental health promotion interventions that exist for children and adolescents are conducted in kindergarten or school, and it is conceivable that mental health promotion interventions should take place in areas where adolescents spend most of their time. Yet, there is another place where adolescents spend much of their spare time, specifically the internet. First and foremost, adolescents spend a lot of their time on social media sites such as YouTube, TikTok, and Instagram [6], but they also use the internet for information-seeking purposes [7]. Using web-based services to provide adolescents with mental health interventions may thus have great potential for preventive health care, as these services can reach a large proportion of youngsters, regardless of where they live. The services are also cost-effective and appeal to young people [8].

Even though young people’s extensive use of social media and online services enables opportunities for socialization, entertainment, and rapid information retrieval, adolescents may also experience negative online events, such as mean comments, exclusion from events and groups, or threats [9]. These events can have severe consequences for adolescents’ well-being [10,11] and are related to serious mental health problems, such as depression, anxiety, and suicidal ideation [9,12,13]. For this reason, there is a need for intervention strategies that can have a reducing effect on negative online events [14] and increase adolescents’ coping skills so that they can better respond to negative online events. However, in Norway and in many other countries, there is a lack of evidence-based interventions to support adolescents who are exposed to negative online incidents [15]. Giving young people access to information on how to deal with negative online events through a mobile app may be a solution that adolescents appreciate.

A quick search on Google Play or the App Store shows that new apps that aim to improve young people’s mental health are constantly being developed. However, the apps that are on the market are of varying quality [16], and there is a broad agreement that more research is needed into the effect of mental health apps and the extent to which they can be a useful tool for mental health promotion and prevention among adolescents [17-19]. It is therefore important that the mental health interventions delivered through apps are evidence-based and of high quality. In Norway, the Regional Centre for Child and Youth Mental Health in the North has developed 2 mental health–promoting mobile apps for adolescents aged 11 to 19 years, called Opp and NettOpp. The apps are educational and informative self-help tools that can support and help young people who face various challenges. The aim of this study is to examine how adolescents evaluated Opp and NettOpp through a user survey.

### Opp

**Figure 1:**
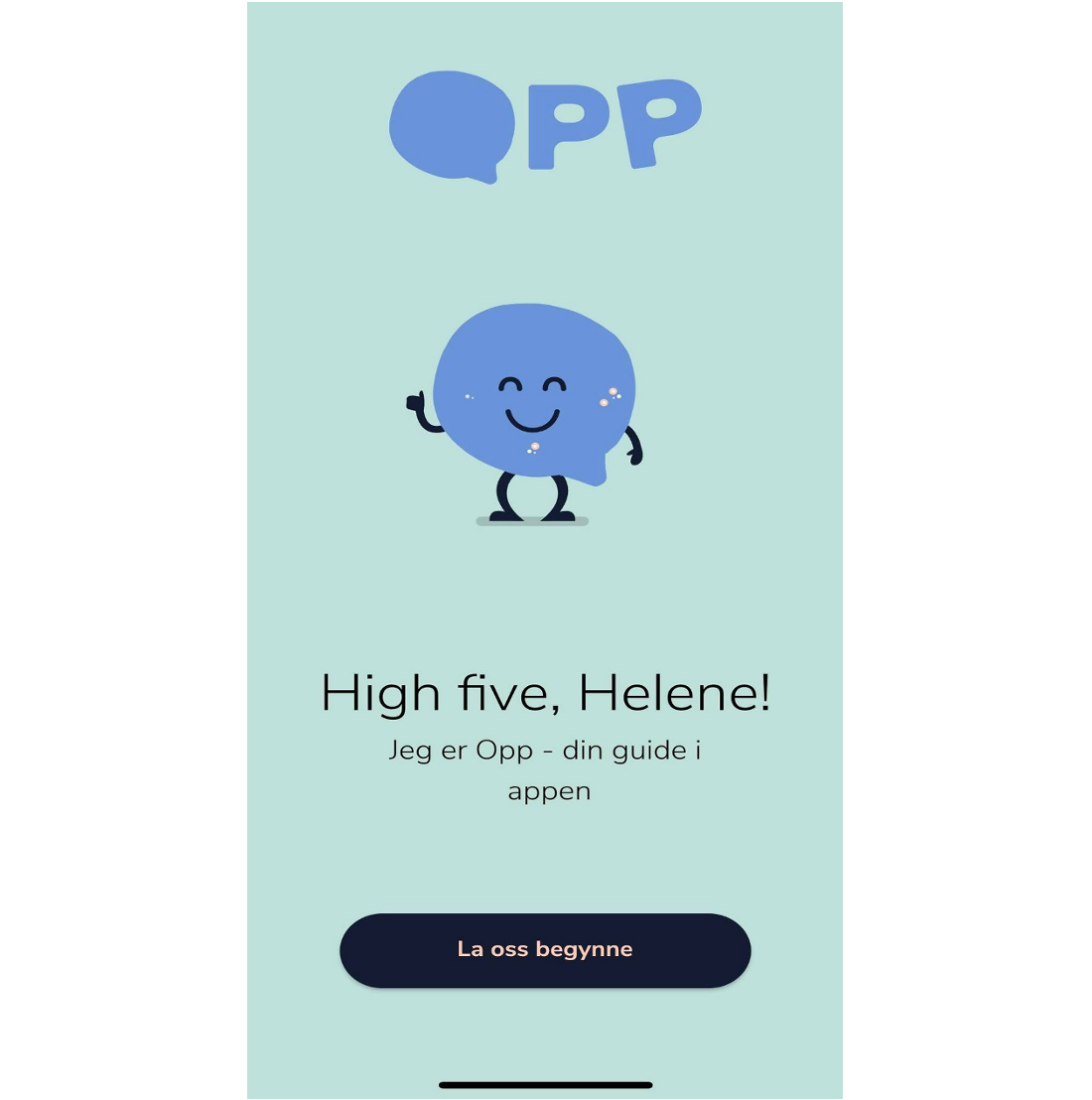
Start page of Opp.

Opp (Figure 1) is a universal mental health–promoting mobile app for adolescents aged between 13 and 19 years. The app was developed in collaboration with adolescents in the target group as well as professionals who work with young people and mental health (ie, nurses, psychologists, and social workers). The overall aim of Opp is to increase adolescents’ well-being, mental health, and coping skills so that they can better cope with stress. The app also aims to reduce mental health problems among adolescents. The app consists of 2 modules. The first module is psychoeducational, with information about mental health and feelings and details of where adolescents can seek help (eg, parents, teachers, or online resources such as the Red Cross and the emergency telephone line for children and adolescents). The second module is a resource module that provides information, exercises, and techniques for how to better cope with stress and difficult thoughts and emotions. These exercises include breathing exercises (visual breathing animation), sound-recorded sleep hygiene advice, mindfulness and mentalizing exercises, and exercises inspired by cognitive behavioral therapy (recognizing and challenging negative thoughts). Information is displayed in the app through text, sound recordings, and animations.

### NettOpp

**Figure 2:**
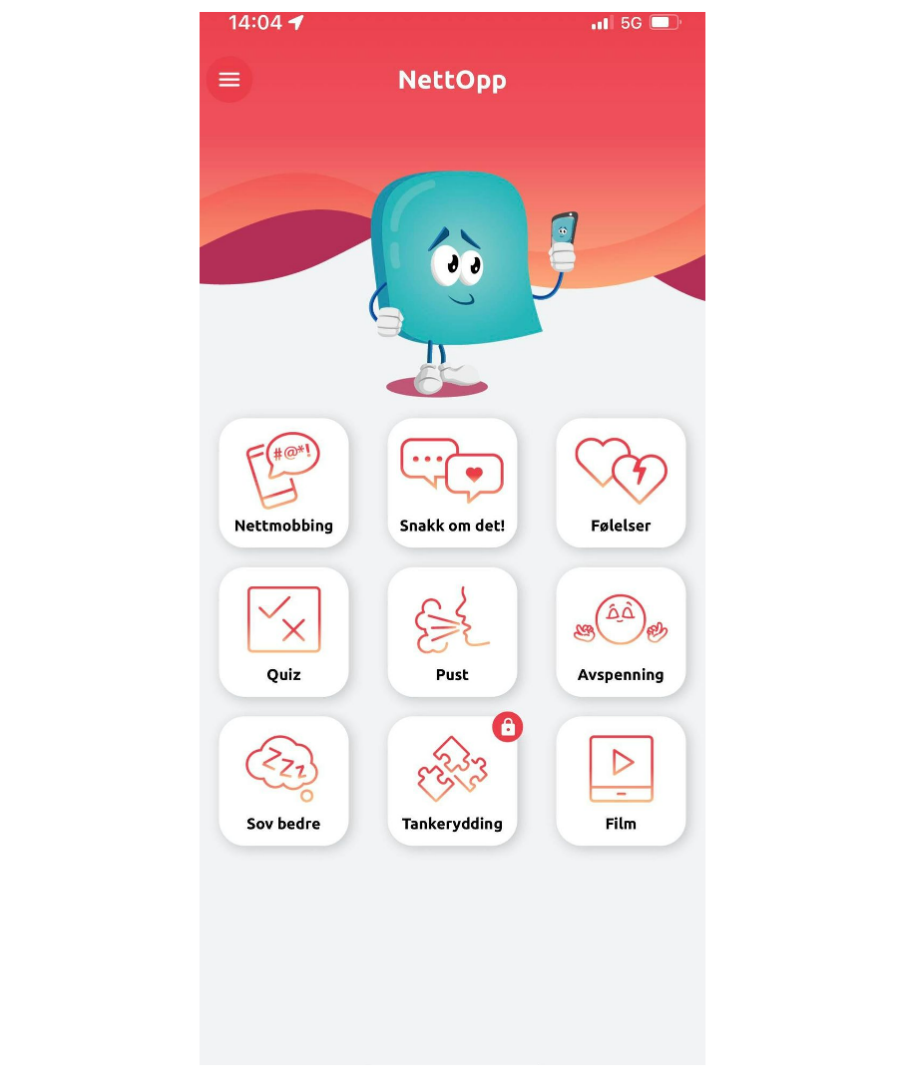
Start page of NettOpp.

NettOpp (Figure 2) is a mobile app designed for adolescents aged between 11 and 16 years to help them cope with negative online events. The app was developed in collaboration with adolescents in the target group as well as professionals who work with young people and bullying (ie, teachers, school health nurses, community psychologists, and the local antibullying professional). The overall aims of NettOpp are to reduce distress related to negative online incidents (eg, cyberbullying) and to increase adolescents’ knowledge of and ability to deal with adverse events online. NettOpp consists of 2 modules. The first module is psychoeducational, with information about negative online incidents (eg, cyberbullying), emotions one may experience related to adverse online events, and information about where and how to seek help. The other module is a resources module, which provides both exercises and stress-related coping techniques. The intervention is described in Kaiser et al [20].

### Objective of This Study

This study examines how adolescents evaluate the usability and quality of Opp and NettOpp, as well as their potential to achieve their given objectives. The study aims to respond to the following research questions:

- RQ1: Are young people satisfied with the system usability of Opp and NettOpp?
- RQ2: How do young people assess the quality of Opp and NettOpp?
- RQ3: How do young people assess Opp and NettOpp’s potential to achieve their goals?

## Methods

### Participants and Procedure

A total of 45 adolescents within the age range of 11 to 19 years were recruited in 2021 to participate in 2 cross-sectional user surveys. The participants were recruited through the authors’ colleagues and acquaintances. A total of 30 adolescents (n=20 girls and n=10 boys) evaluated Opp, and 15 adolescents (n=6 girls and n=9 boys) evaluated NettOpp. For Opp, 3 (10%) adolescents stated that they had not used the app at all and were therefore excluded from the analyses. Hence, the results presented are based on 27 adolescents (n=8 boys and n=19 girls) evaluating Opp. After a trial period of approximately 3 weeks, the adolescents who evaluated Opp answered an online questionnaire in Nettskjema [21], while the adolescents who evaluated NettOpp answered a paper-based questionnaire.

### Measures

#### System Usability

The usability of the apps was measured using the System Usability Scale (SUS) [22] with modified SUS statements [23]. The scale consists of 10 items (eg, “I thought the product was easy to use” and “I found the product unnecessarily complex”). Responses were rated on a 5-point scale from 0 (*strongly disagree*) to 4 (*strongly agree*). SUS scores were summarized before being multiplied by 2.5 to transform the SUS score into a 0 (*low perception of usability*) to 100 (*high perception of usability*) interval scale. SUS scores may be interpreted using school-grade ratings. The guidelines for interpreting the SUS scores are D, which ranges from 60 to 70, C from 70 to 80, B from 80 to 90, and A from 90 to 100 [24]. The SUS was translated into Norwegian by the second and third authors. The adolescents could also comment on the apps’ system usability in a supplementary open comment field at the end of the questionnaire.

#### App Quality

The quality of the apps was assessed with items from the quality rating section of the Mobile Application Rating Scale (MARS) [25]. Originally, the MARS’ quality rating section consisted of 23 items divided into 4 subscales to assess engagement (section A), functionality (section B), aesthetics (section C), and information (section D), and 1 subscale to assess subjective quality ratings (section E). To adapt the scale to adolescents, we only included questions that we anticipated were easy to answer for young people and that we believed would give us a good indication of important aspects of the quality of the apps. Hence, we included and adapted 10 items from the original scale, of which 7 items are from sections A-D (eg, “How accurately do the app’s features and components work?”) and 3 items are from section E (eg, “What is your overall star rating of the app?”). All items were rated on a 5-point scale with different response categories from 1 (*App is broke*n and *I would not recommend this app to anyone*) to 5 (*Perfect/timely response* and *one of the best apps I’ve used*)*.* The adolescents could also comment on the apps’ quality in a supplementary open comment field at the end of the questionnaire.

### Potential Goal Achievement

To measure how the adolescents evaluated the apps’ potential to achieve their given objectives, the app-specific rating section from the MARS [25] was used for the adolescents who evaluated NettOpp. The app-specific section in MARS consists of 6 items that can be used to access the perceived impact of an app on users’ awareness, knowledge, attitudes, intentions to change, and the likelihood of actual behavior change. The items were adjusted to match the target health behavior for the app. Example items for NettOpp are “NettOpp is likely to increase awareness of the importance of addressing cyberbullying” or “The use of NettOpp is likely to encourage seeking further help after being exposed to cyberbullying.” The adolescents who evaluated Opp were given more adapted questions (inspired by the app-specific section in MARS) about each health goal of the app. Example items from Opp are “Opp is likely to increase well-being and mental health” or “Opp is likely to motivate young people to speak out or ask for help when they need it.” The adolescents rated their answers on a 5-point scale from 1 (*strongly disagree*) to 5 (*strongly agree*). The adolescents could also comment on the apps’ potential goal achievement in a supplementary open comment field at the end of the questionnaire.

### Ethics Approval

The evaluation studies were approved by the Norwegian Centre for Research Data (reference for Opp: 450518; reference for NettOpp: 545417). Active parental consent from 1 guardian was required for the adolescents to participate in the study. The consent was retrieved in a digital consent form using Nettskjema [21], where the guardian declared that they understood the purpose of the study and what was required of their child if they participated. The adolescents’ answers were collected anonymously. All participants were compensated with a cinema gift card with a value of NOK 150 (US $15.03). This study did not need approval from the Regional Committees for Medical and Health Research Ethics as no health-related information was collected.

### Statistical Analyses

All statistical analyses were performed in SPSS version 27 (IBM Corp). Descriptive statistics were calculated and included means, standard deviations, and frequency distributions. An independent sample *t* test was conducted to investigate the difference in the overall star evaluation of the apps between Opp and NettOpp users.

## Results

### System Usability of Opp and NettOpp

The overall mean SUS scores of Opp and NettOpp were 80.37 (SD 9.27) and 80.33 (SD 10.30), respectively. An overview of adolescents who agreed with statements in the SUS is presented in Table 1. None of the adolescents who evaluated Opp (0/27, 0%) reported that the app was difficult to use; 4% (1/27) stated that they needed to learn a lot before they could use Opp, and 33% (9/27) thought that they would use the app frequently. Furthermore, none of the adolescents (0/15, 0%) who evaluated NettOpp stated that the app was difficult to use, 33% (5/15) reported that they would use the app frequently, and approximately 7% (1/15) stated that they needed to learn a lot before they could use the app.

**Table 1:** Percentage of adolescents who answered that they strongly agree or agree with claims about NettOpp and Opp’s system usability.

	Opp (n=27), n (%)	NettOpp (n=15), n (%)
I think I would like to use this product frequently	9 (33)	5 (33)
I found the product unnecessarily complex	1 (4)	0 (0)
I thought the product was easy to use	25 (93)	13 (87)
I think that I would need the support of a technical person to be able to use this product	0 (0)	0 (0)
I found the various functions in the product were well integrated	22 (82)	14 (93)
I thought there was too much inconsistency in this product	0 (0)	0 (0)
I imagine that most people would learn to use this product very quickly	26 (96)	15 (100)
I found the product very awkward to use	2 (7)	0 (0)
I felt very confident using the product	19 (70)	11 (73)
I needed to learn a lot of things before I could get going with this product	1 (4)	1 (7)

### The Quality of Opp and NettOpp

Descriptive results from the adolescents’ evaluation of the quality of the apps are presented in Table 2. A large proportion of the adolescents thought that Opp was interesting to use (20/27, 74%), that the app functioned well (26/27, 96%), that the visual appeal was good (25/27, 93%), and that the app was appropriate for the target audience (24/27, 89%). Furthermore, a large proportion of the adolescents thought that NettOpp was interesting to use (12/15, 80%), that the app content was appropriate for the target group (14/15, 93%), and that the visual appeal was good (12/15, 80%). They also stated that they would recommend NettOpp to anyone who might benefit from it (13/15, 87%). There was a statistically significant difference in the overall star evaluation between adolescents testing NettOpp (mean 4.20, SD 0.56) and Opp (mean 3.78, SD 0.42; t_40_=2.75; *P*=.01).

**Table 2:** Descriptive statistics of the quality ratings for the mobile apps NettOpp and Opp.

	Opp (n=27)		NettOpp (n=15)	
	Min-max	Mean (SD)	Min-max	Mean (SD)
Is the app interesting to use?	3-5	3.78 (0.51)	3-5	4.00 (0.65)
Is the app content appropriate for the target audience?	3-5	4.37 (0.68)	3-5	4.47 (0.64)
How accurately or fast do the app features and components work?	3-5	4.67 (0.55)	1-5	4.13 (1.25)
How easy is it to learn how to use the app?	3-5	4.48 (0.65)	3-5	4.53 (0.64)
Is arrangement and size of buttons or icons (…) on the screen appropriate or zoomable?	2-5	4.37 (0.79)	4-5	4.53 (0.52)
How good does the app look?	2-5	4.15 (0.66)	3-5	4.13 (0.74)
Does the app contain what is described?	3-5	4.26 (0.17)	4-5	4.80 (0.41)
Would you recommend this app to people who might benefit from it?	2-5	3.68 (0.84)	1-5	4.33 (0.74)
How many times do you think you would use this app in the next 12 months?	1-5	3.33 (1.04)	1-5	3.47 (1.25)
What is your overall star rating of the app?	3-4	3.78 (0.42)	3-5	4.20 (0.56)

### Potential Goal Achievement of Opp and NettOpp

Tables 3 and 4 provide an overview of the percentages of adolescents who agreed with statements regarding Opp and NettOpp’s ability to achieve their goals, respectively. A large percentage agreed that Opp would increase knowledge about mental health (25/27, 92%) and help young people deal with stress, difficult emotions, or situations (18/27, 67%). Furthermore, 52% (14/27) thought Opp would increase well-being and mental health (11/27, 41% agreed to some extent), 37% (10/27) thought that Opp would reduce mental health problems among adolescents (14/27, 52% agreed to some extent), and 33% (9/27) thought Opp would motivate young people to seek help if they needed it (16/27, 59% agreed to some extent). A large percentage (n=11-15, 73%-100%) of the 15 adolescents who evaluated NettOpp agreed that the app could increase awareness and knowledge about cyberbullying, attitudes toward cyberbullying, and motivation to address cyberbullying. Only 33% (5/15) thought that NettOpp could reduce cyberbullying among adolescents.

**Table 3:** Proportion of adolescents answering that they strongly agree or agree with statements about Opp’s potential to achieve its goals.

Opp will probably...	Participants (n=27), n (%)
Increase well-being and mental health	14 (52)
Increase knowledge about mental health	25 (92)
Help young people deal with stress, difficult emotions, or situations in a good way	18 (67)
Motivate young people to speak out or ask for help when they need it	9 (33)
Make young people feel better about themselves	14 (52)
Decrease mental health problems	10 (37)

**Table 4:** Proportion of adolescents who answer that they strongly agree or agree with statements about NettOpp’s potential to achieve its goals.

NettOpp will probably...	Participants (n=15), n (%)
Increase awareness of the importance of addressing cyberbullying	15 (100)
Increase knowledge and understanding of cyberbullying	14 (93)
Change attitudes toward improving reactions of cyberbullying	11 (73)
Increase intentions or motivation to address cyberbullying	13 (87)
Encourage further help seeking after being exposed to cyberbullying	14 (93)
Decrease cyberbullying	5 (33)

### User Comments on the Apps’ Usability and Quality

In total, 7 adolescents left a comment about Opp and 5 left a comment about NettOpp in the open comment field (all comments are translated and presented in Textbox 1). The comments from the young people who tested Opp concerned design, app functions, and quality. A total of 3 adolescents who left a comment about NettOpp stated that the app had crashed or shut down while in use.

**Supplementary comments from the adolescents in both the Opp and NettOpp samples.**

**Opp**

- I will not use the app again because I currently have nothing that bothers me and I bet it will stay that way, but I will probably use it if I start to struggle mentally.
- I really like that the little figure praises you when you finish an exercise.
- The app is probably best suited for children who are 10-14 years old, for example, if they struggle to talk to their parents. If the app is intended for all adolescents, the app must be adapted to older adolescents as well.
- Maybe a little too much text but that is fine.
- Very nice app! But I think it was difficult to get an overview of the main menu and what the app contained. It seemed more like you pressed something, and you also moved on, but it was difficult to come back.
- All the exercises with audio files are very well formulated and informative, but difficult to use. The biggest problem is that you cannot listen to the audio file while the phone is in sleep mode (the screen is off). It was disturbing for the experience of the exercises when there was an interruption since the mobile phone automatically switches off and the sound from Opp is cut off. Functions such as listening to sounds such as music or podcasts in Spotify, etc is desired – so you can listen to the audio file or videos even if the phone automatically shuts off the screen.
- There could have been more exercises, for example, relaxation exercises.
- Feeling empty should have been an alternative, missed it when I was tired and did not really feel anything.

**NettOpp**

- The app was easy to use. I never needed help from my parents to use the app. The quiz was good. Breathing technique exercise was good. I have tested them several times.
- The app was very user-friendly. Very good to point out that experiencing cyberbullying is not your fault. The app’s main menu may possibly feel a bit childish, but I liked the layout.
- I think it is a very good app with many features that are easy to use. Even though I do not need it, I think those who use it will be happy.
- A bit too much text and will think that for some it will take away the motivation, and they cannot bear to read everything. Still feels that everything that is written is relevant. Technical problem: The app automatically closes several times while in use. I can go into it right afterward and it works as it should, but after a while is “thrown out” again. I think there is a nice common thread throughout the app, and I like the suggestions that come up along the way.
- The menu button in the left corner can be a little more visible, it was a little difficult to see. Once you have taken the quiz, you will not see results, you can only choose to return to previous questions or close the page. Had been nice to see results and get a “prize.” The test app crashes all the time. The lock icon on thought clearing was confusing and made me think that the function was locked and that I could not use it. Very good design and nice app. Very good idea.
- Some words were difficult, the app crashes 3 times, PLZ fix it!
- Try to fix the piece where you are thrown out.

## Discussion

### Overview

Several health-promoting apps have been developed in recent years, but only a few of them have been evaluated. Furthermore, studies that evaluate mobile apps are of varying quality [16]. This study is a user survey of 2 mental health-promoting apps, called Opp and NettOpp. Although a user survey does not support conclusions about the effectiveness of the apps in terms of goal achievement, it does provide important information about how adolescents evaluate the apps’ usability and their overall quality. Since online interventions have varying reported effects on adolescents’ mental health [17], it is also interesting to know more about what adolescents think a specific app can help them with. In this study, young people quality-assured the 2 mobile apps NettOpp and Opp, which are intended to promote mental health among adolescents.

### Principal Results

Overall, the adolescents were satisfied with the system usability of both Opp and NettOpp. SUS scores for Opp and NettOpp corresponded to a B grade on the grade scale and to “excellent” for the adjective ratings [23]. Usability can be defined as the extent to which *“a product can be used by specified users to achieve specified goals with effectiveness, efficiency, and satisfaction in a specified context of use”* [26]. A SUS score above 70 is considered acceptable [23]. Previous research has found an average SUS rating of 77.3 for the 10 most popular apps, such as Facebook, YouTube, and iTunes [27], where mobile apps overall had greater usability than apps adapted to other devices, such as tablets. An examination of each item on the SUS in this evaluation study showed broad agreement among the adolescents who evaluated Opp that the app was easy to use and that most people would learn how to use the app. Furthermore, the adolescents who evaluated NettOpp agreed that the app was easy to use, that the functions were well integrated, and that most people would learn how to use it. A smaller proportion of the adolescents who tested the apps thought that they would use Opp and NettOpp regularly themselves.

There was also overall agreement among both the adolescents who evaluated Opp and those who evaluated NettOpp that the apps were of good quality. In this study, only some of the items from MARS were included in the questionnaire, so it will not be possible to compare the results of this evaluation study with previous studies using the complete MARS quality ratings [28-30]. However, previous research has shown that adolescents emphasize the importance of mental health apps being engaging for young people as well as having a useful and relevant function [31]. In this evaluation study, most of the adolescents stated that both apps were interesting to use and that the content was appropriate for the target group. The adolescents also stated that the apps’ visual appeal was good. In the overall evaluation of the apps, NettOpp received a higher ranking than Opp. Although it is difficult to draw conclusions about the practical implications of this finding, we expected that the adolescents would prefer Opp to NettOpp. NettOpp is more static and has a simpler design, while Opp is more complex, with more functions, animations, and exercises in addition to a more sophisticated design. Kenny et al [31], on the other hand, found that adolescents appreciate apps that are concise and easy to navigate. In addition, study participants commented that Opp was difficult to navigate. Another reason for the higher ranking of NettOpp might be that this app is specifically targeted at adolescents who might have challenges related to negative online events, while Opp is a universal mental health–promoting mobile app for all adolescents. NettOpp might therefore be perceived as more helpful.

As both apps aim to promote the mental health of adolescents, we sought to assess the adolescents’ opinion of whether the apps would have the ability to achieve their given objectives. Among the adolescents who evaluated Opp, there was broad agreement that the app would increase adolescents’ knowledge of mental health. Previous research has shown that teaching young people about mental health may be an effective preventive measure, which can also contribute to reducing the stigma related to mental disorders [32,33]. Previous studies have also shown that strengthening adolescents’ knowledge of mental health may increase help-seeking behavior [34,35]. Motivating adolescents to seek help when they need it is important because it can prevent the development of disorders through early interventions. This evaluation study showed that a large proportion of the adolescents agreed to some extent that Opp could motivate adolescents to speak out or ask for help when they needed it. However, a smaller proportion of the sample thought Opp could reduce mental health problems among adolescents. This may indicate that the adolescents see mental health problems as a more complex problem that cannot be solved, for example, by increasing the adolescents’ knowledge or motivating them to seek help.

The sample that tested NettOpp thought that the app would increase awareness and knowledge of negative online incidents. Previous research has illustrated the importance of increasing knowledge and awareness about negative online incidents to ensure that both adolescents and people who work closely with the group (eg, teachers) are capable of handling the issue in an effective way [36]. Furthermore, previous research shows that adolescents who experience negative online incidents are often reluctant to seek help [37,38]. As adolescents who experience negative online incidents more often experience mental health issues, it is important to motivate them to seek help. In this evaluation study, the adolescents thought NettOpp would increase help-seeking behavior among adolescents when they experienced negative online incidents. There was, however, a small percentage of the sample who thought NettOpp would reduce negative online incidents. Previous research supports the youth’s evaluation regarding the aim of reducing negative online incidents, as this is a comprehensive problem and simple intervention is not enough to change it [39]. Reducing cyberbullying often requires extensive cooperation between various agencies, such as school workers and parents. However, as cyberbullying can have severe consequences for adolescents’ mental health and interventions to prevent psychological distress are greatly needed, NettOpp may be a helpful tool. The adolescents’ evaluations indicate that NettOpp can give them access to information to increase young people’s coping skills when they face negative online incidents, and this may be an effective preventive measure.

### Limitations

Important limitations to address are related to the sample sizes and the sample itself that evaluated the apps, as well as to the cross-sectional design of the study. The 2 sample sizes were relatively small and not representative, so the findings of this study cannot be generalized to all adolescents in Norway. However, for the purpose of this study, we think that the sample sizes were sufficient. Some of the adolescents who evaluated the apps were also involved in developing the apps. This might have influenced their assessment in that they might, for example, have been more positively tuned when answering the questions. However, since study participation was anonymous, we cannot examine if it actually influenced the participants. The participants in the evaluation study were also compensated with a cinema gift card with a value of NOK 150 (US $15.03), which may have influenced the motivation of the study participants. Further, the age range of the sample who evaluated the apps was between 11 and 19 years. NettOpp’s target group is children and adolescents between the ages of 11 and 16 years, and Opp’s target group is adolescents aged between 13 and 19 years. One of the adolescents left a comment saying that Opp was best suited for adolescents aged between 10 and 14 years, and another adolescent left a comment saying that NettOpp was a bit childish. These comments may be due to the fact that the evaluators were older than the intended target group. However, the adolescents’ exact ages were not collected, which is a limitation for both this study and further work with the app. Another possible explanation is that some young people consider the design of the apps to be childish because they are more sophisticated than the designers and developers had anticipated.

As the aim of the study was to examine how adolescents assess the usability and quality of 2 mental health mobile apps, a cross-sectional design was justified as it allowed for an impression of a given topic at a given time point. However, a cross-sectional design does not allow for drawing conclusions about the long-term use of the apps or about their actual effectiveness.

Another limitation of this evaluation study is related to the apps’ functionality during the trial period. Comments were received for both Opp and NettOpp. Some adolescents found it difficult to navigate Opp. Several of the adolescents who evaluated NettOpp reported that the app crashed while they were using it. This is a frequent problem reported among app users [40]. It is therefore possible that these problems affected the adolescents’ experience with the 2 apps. However, because of the feedback on NettOpp’s functional problems, the app was updated and fixed.

A small proportion of adolescents who tested Opp thought the app could reduce mental health problems among adolescents. Only a few of the adolescents who evaluated NettOpp thought the app could reduce negative online incidents. It was not controlled in either the Opp sample or the NettOpp sample whether the adolescents had experienced mental health issues or negative online incidents. This was because we were mainly interested in mapping app usability and quality before they were evaluated in wider effectiveness studies.

### Conclusions

This user evaluation study has provided helpful and important knowledge about how adolescents assess the apps’ usability, quality, and potential to achieve their given objectives. Overall, the findings are positive and promising. They suggest that mental health–promoting mobile apps offer a practical, relatively cheap, and appealing approach to reaching adolescents and improving their mental health. However, the cross-sectional design of the study does not allow for drawing any conclusions about the long-term use of such apps among adolescents. It is therefore necessary to conduct further studies that examine the use of mental health–promoting mobile apps in the long run. This study does not provide answers about the effectiveness of the 2 apps. It is therefore also necessary to conduct wider effectiveness studies for both apps in order to draw any conclusions on their actual goal achievement.

## Abbreviations

MARS: Mobile Application Rating Scale
SUS: System Usability Scale

## Data Availability

The data sets generated during or analyzed during this study are available from the corresponding author on reasonable request.
